# Simulation of small-angle X-ray scattering data of biological macromolecules in solution

**DOI:** 10.1107/S1600576720000527

**Published:** 2020-02-18

**Authors:** Daniel Franke, Nelly R. Hajizadeh, Dmitri I. Svergun

**Affiliations:** a European Molecular Biology Laboratory, Notkestrasse 85 c/o DESY, Hamburg, 22607, Germany; b ETH, ScopeM IDA Group, HPZ G 35.1, John-von-Neumann-Weg 9, 8093, Zurich, Switzerland

**Keywords:** small-angle X-ray scattering, SAXS, data simulation, computer programs, solution scattering

## Abstract

This article presents *IMSIM*, an application to simulate two-dimensional small-angle X-ray scattering patterns and, further, one-dimensional profiles from biological macromolecules in solution. *IMSIM* implements a statistical approach yielding two-dimensional images in TIFF, CBF or EDF format, which may be readily processed by existing data-analysis pipelines. Intensities and error estimates of one-dimensional patterns obtained from the radial average of the two-dimensional images exhibit the same statistical properties as observed with actual experimental data. With initial input on an absolute scale, [cm^−1^]/c[mg ml^−1^], the simulated data frames may also be scaled to absolute scale such that the forward scattering after subtraction of the background is proportional to the molecular weight of the solute. The effects of changes of concentration, exposure time, flux, wavelength, sample–detector distance, detector dimensions, pixel size, and the mask as well as incident beam position can be considered for the simulation. The simulated data may be used in method development, for educational purposes, and also to determine the most suitable beamline setup for a project prior to the application and use of the actual beamtime. *IMSIM* is available as part of the *ATSAS* software package (3.0.0) and is freely available for academic use (http://www.embl-hamburg.de/biosaxs/download.html).

## Abstract 2

An application is presented to simulate images on an area detector for isotropic small-angle X-ray scattering, explicitly accounting for the experimental geometry and yielding radially averaged 1D scattering patterns with statistically appropriate variations.

## 1. Introduction   

A small-angle X-ray scattering (SAXS) experiment performed on a solution of biological macromolecules typically produces an isotropic 2D scattering pattern originating from the random orientations of the molecule in solution. To access shape and size information of the solute, the image is first radially (or azimuthally) averaged into a 1D curve, which depicts the scattering intensity *I*(*s*) against the momentum transfer *s*. The scattering intensities depend on the wavelength (λ) and the angle between the incoming and scattered photons (2Θ) as *s* = (4πsinΘ)/λ. To extract the structural parameters of the molecule, the background contribution to the scattering from components such as the capillary or the buffer needs to be subtracted from that of the sample.

So far, the most common approach to calculate SAXS data has been at the level of the 1D curve, typically on the basis of atomic structures (Svergun *et al.*, 1995 ▸; Schneidman-Duhovny *et al.*, 2010 ▸; Knight & Hub, 2015 ▸; Grishaev *et al.*, 2010 ▸; Liu *et al.*, 2012 ▸; Tjioe & Heller, 2007 ▸; Virtanen *et al.*, 2011 ▸) or from geometric bodies (Konarev *et al.*, 2003 ▸). There are significantly fewer options for generating 2D scattering images. Of those available, most are tailored to calculate the scattering from oriented systems and/or for single-particle diffraction experiments, both for coherent and incoherent sources, using explicit equations or numerical methods (Chen & Luo, 2017 ▸; McAlister & Grady, 2002 ▸; Fritz-Popovski, 2013 ▸; Saldin *et al.*, 2010 ▸; Alves *et al.*, 2017 ▸). All these latter methods result in theoretically calculated 2D images or 1D scattering patterns without any random components. For simulations of experimental data, approximations of random noise are often added by *ad hoc* methods.

As many aspects of the beamline setup, *e.g.* detector type, sample–detector distance, X-ray wavelength and the incident beam position on the detector, are propagated into the uncertainties of the final 1D scattering pattern, it is necessary to simulate scattering patterns on area detectors. Importantly, the instrumental parameters influence not only the angular range and spacing but also the point-to-point variation of the individual data points in the 1D scattering data. Here we present *IMSIM*, an application to accurately simulate a 2D SAXS scattering image based on the input of a calculated 1D SAXS curve: with the focus that, when radially averaged again, it accurately reproduces the source data up to a scaling factor and the desired random noise.

An overview of all command-line options and arguments of *IMSIM*, as well as a full usage example, are provided online: https://www.embl-hamburg.de/biosaxs/manuals/imsim.html.

## 2. *IMSIM* architecture   

### 2.1. Simulation method   

**Figure 1:**
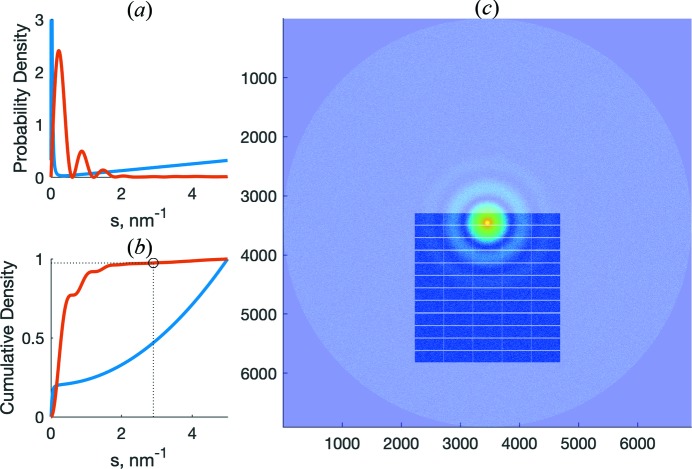
Simulation of native horse spleen ferritin (PDB code 1ier; Granier *et al.*, 1997 ▸) at 6.0 m detector distance. The theoretical scattering pattern was calculated by *CRYSOL* (Svergun *et al.*, 1995 ▸) with 5000 points, in the range from 0.0 to 5.0 nm^−1^. (*a*) The expected probability density for the simulated events of the flat background (blue) and of the sample (red), adjusted for the increase in the detector area. (*b*) The corresponding cumulative density functions used to determine the non-uniform *s* position from a uniform random number in [0, 1]. (*c*) The resulting image at a 6.0 m position with a Pilatus 6M detector mask applied; here the count data are displayed on a logarithmic scale for improved visualization.

The simulation is initialized with a continuous rectangular virtual detector plane of infinite size in polar coordinates. Individual random photon-like arrival events are generated in polar coordinates as well, then mapped and quantized onto a rectangular pixel grid, such that the resulting Cartesian image exhibits the same statistical properties and overall features as observed with experimental data. Here, sampling in polar coordinates is a canonical choice as isotropic scattering implies no preference of direction; hence a random variable of uniform distribution over [−π, π) may be employed to determine the direction. In turn, the probability of a certain distance is proportional to the intensities of the calculated input scattering scaled by the Cartesian circle area and normalized to sum to 1.0 [Fig. 1 ▸(*a*)]. Drawing randomly from a uniform variable over the interval [0, 1] and applying the quantile function of the intensity probability distribution yields the angular position of the virtual photon event [Fig. 1 ▸(*b*)]. The total number of overall events generated depends on the input parameters on application start-up, *e.g.* virtual flux, exposure time and concentration. Each random set of polar coordinates is then mapped to Cartesian space and, in the last step, quantized to the configured detector pixel grid with a point-spread function of one pixel. The physical dimensions of the final image depend on the wavelength, sample–detector distance and pixel size of the virtual detector [Fig. 1 ▸(*c*)].

### 2.2. *IMSIM* input/output   

*IMSIM* is a command-line application that takes as input a file with calculated one-dimensional scattering intensities on an absolute scale (Svergun *et al.*, 1995 ▸), with the first and second columns corresponding to the momentum transfer in inverse ångström and calculated intensities, respectively. To simulate the 2D scattering pattern, setup details such as the detector type, exposure time (seconds), X-ray wavelength (metres), detector distance (metres) and flux (events per second) need to be specified through command-line options. There are currently two types of detectors pre-defined in *IMSIM*, namely Pilatus and Eiger photon-counting detectors of various sizes (Broennimann *et al.*, 2006 ▸; Dinapoli *et al.*, 2011 ▸). Here, the detector selection defines the physical dimensions as pixel count and size of the final image. Non-photon-counting detectors, *e.g.* CCDs, are not (yet) implemented in *IMSIM* as they have a point-spread function larger than the one pixel of the Pilatus and Eiger detectors. Introducing a point-spread function would be conceptually simple and could be done on popular request. The sample concentration has to be provided as well, where a concentration of 0.0 corresponds to simulation of background scattering only. Analogous to the sample scattering, a background scattering pattern on an absolute scale may be supplied to be used as background, for example scattering of a filled capillary. Otherwise a default ‘flat’ background modeled by a double-exponential function^1^ is applied.

For convenience, *IMSIM* also provides means to obtain a template for a beamstop mask with module gaps suitable for the selected detector and the angular axis for the defined experimental setup. The axis file is generated using information about the wavelength and sample–detector distance, while the shape of the mask is defined by the detector geometry (*e.g.* size and positions of the detector modules). Masks may be exported in a *FIT2D* binary .MSK format (Hammersley, 2016 ▸) and it is possible to shift the mask on the 2D scattering pattern by defining offsets in the **x** and **y** directions to account for different locations of the beam center on the 2D image [Fig. 1 ▸(*c*)]. The default output of *IMSIM* is an image with the simulated 2D scattering pattern in TIFF, EDF or CBF format.

### 2.3. Statistical properties   

**Figure 2:**
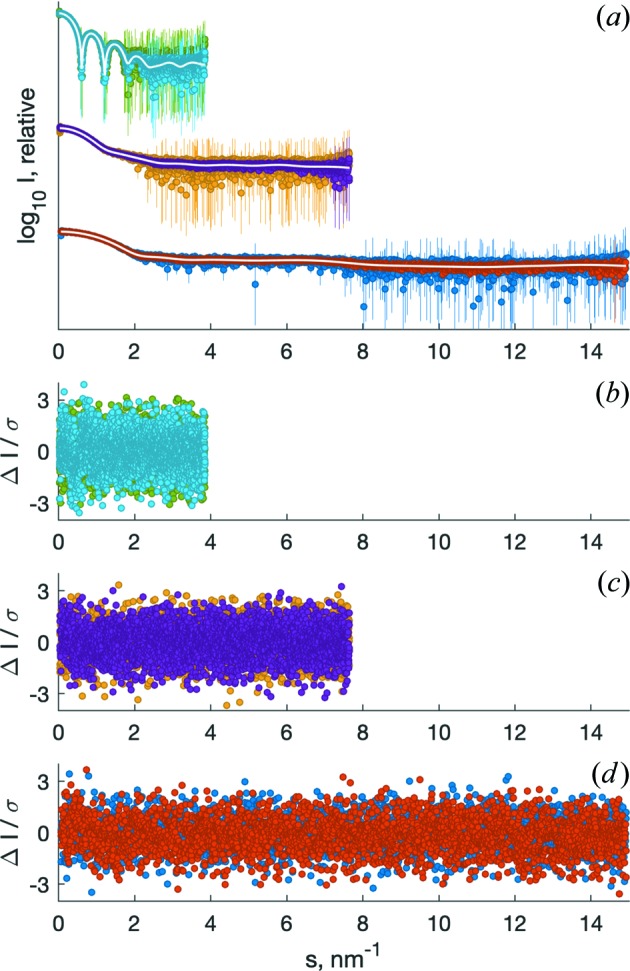
Examples of simulated data from samples of varying size and shape with constant wavelength at multiple distances of the Pilatus 6M detector from the sample position. All backgrounds were generated with an exposure time of 10 s, the samples with an exposure of 1 s. (*a*) The simulated data of three different samples after background subtraction and scaling by concentration, offset for clarity. These are native horse spleen ferritin (1ier) at 6.0 m detector distance at a concentration of 0.5 mg ml^−1^ (green) and 2 mg ml^−1^ (light blue); bovine serum albumin (4f5s; Bujacz, 2012 ▸) at 3.0 m detector distance at a concentration of 0.5 mg ml^−1^ (yellow) and 2.0 mg ml^−1^ (violet); and myoglobin (1wla; Maurus *et al.*, 1997 ▸) at 1.5 m detector distance at a concentration of 0.5 mg ml^−1^ (dark blue) and 2.0 mg ml^−1^ (red). The calculated scattering curves are overlaid in white. Error bars of all data are relative errors in logarithmic scale {SE(*I*)/[*I*log(10)]} and partially truncated for visibility. (*b*)–(*d*) The respective standardized residuals of the simulated data minus the calculated input scattering, divided by the error estimate. Standardized residuals are expected to be randomly distributed and to follow a standard normal distribution, *i.e.* >99% of all standardized residuals should be located in the range [−3; +3].

As presented in Fig. 2 ▸, the 1D scattering patterns after radial averaging with *RADAVER*/*IM2DAT* (*RADAVER* in *ATSAS 2.8* and earlier, *IM2DAT* as of *ATSAS 3.0*; Franke *et al.*, 2017 ▸), background subtraction and normalization are similar to the calculated input data up to the simulated noise. In particular, the standardized residuals are randomly distributed and follow a standard normal distribution. This may be statistically validated with (*a*) the reduced χ^2^ statistic (Pearson, 1900 ▸) to test the hypothesis whether the observed differences between calculated input scattering and simulated output data may be explained by chance alone, *i.e.* whether they are similar up to noise or systematically different; and (*b*) the Anderson–Darling statistic (Anderson & Darling, 1954 ▸; Stephens, 1974 ▸) to test whether the empirical distribution of the standardized residuals is similar to a standard normal distribution with mean zero and standard deviation of one. As detailed in Table 1 ▸, none of the hypotheses of similarity up to noise and standard normal distribution of residuals may be rejected at a significance threshold α = 0.01.

In addition, 1000 independent background frames were simulated and compared pairwise with the reduced χ^2^ test. The obtained histogram of the reduced χ^2^ values of the resulting 499 500 comparisons closely follows the expected χ^2^ distribution (not shown).

## 3. Applications   

*IMSIM* is implemented as a cross-platform command-line application that can easily be used interactively, integrated into shell scripts and also employed as a data source, for example, for testing data-analysis pipelines. As *IMSIM* can quickly generate a large number of realistic scattering patterns from a multitude of setups (Fig. 2 ▸), it may be used for all aspects of method development. Owing to the intentional absence of instrument or sample effects, it is straightforward to validate radial-averaging and data-analysis applications by testing on the simulated data provided by *IMSIM*. Such tests could validate the error propagation, provide insights into the effects of statistical noise on the analysis procedures, or, for example, reveal how over- and/or undersubtraction would influence the results. The methods that so far ignored the information contained in error estimates may be re-evaluated to check whether, or how, accurate error estimates would improve the results. In addition, facility users may use *IMSIM* to evaluate likely experimental outcomes of their system on the basis of the available setup and may request adjustments of detector position or wavelength in advance, if beneficial for the particular specimen. Also, educational facilities may employ *IMSIM* to generate data for courses and presentations and provide them to students who would not have easy access to large-scale synchrotron facilities or laboratory sources to obtain data for training.

## 4. Limitations and future developments   

The method described generates experimentally idealized, but statistically appropriate, data by emulating a virtual beamline with photons being spontaneously created from the provided sample without any physical components that might interfere, *i.e.* no ray-tracing is performed. At present, the sample does not allow for a structure factor coming from specific or unspecific aggregation or repulsion effects in solution. The effects of excluded volume at high solute concentrations are not considered owing to a lack of theoretical background to rely on. Future developments of *IMSIM* may include support for beam profiles, detector point-spread functions and wavelength spread, allowing for the generation of instrumentally smeared (*e.g.* neutron scattering) data.

## 5. Availability   

*IMSIM* is available as part of the *ATSAS* software package (3.0.0; Franke *et al.*, 2017 ▸) and is freely available for academic use (http://www.embl-hamburg.de/biosaxs/download.html).

## 6. Conclusions   

We present a program to simulate isotropic 2D scattering patterns from one-dimensional small-angle X-ray scattering data. The principle is based on simulating the photon arrival on a virtual detector by modeling it as a probabilistic event, generating appropriately randomized data and their errors. As all of the files needed for the radial averaging can be produced by *IMSIM*, *i.e.* mask, axis and uniform background, it is possible to rapidly obtain a subtracted data set based on simulated data that are virtually indiscernible from experimental data. *IMSIM* may be utilized by experimentalists, methods developers and educational facilities in need of a large number of data sets with close resemblance to experimental data.

## Figures and Tables

**Table 1:** Simulation summary and statistics The three samples (exposure time 1 s) are simulated together with a flat background (exposure time 10 s). Each sample is simulated at two concentrations for the given sample–detector distance (different distances are used for all samples). At all positions a 3 mm circular ‘beamstop’ was masked out at the incident beam position, resulting in a different angular range at each detector position. All generated data sets have *n* = 2647 data points. Reduced χ^2^ and Anderson–Darling test statistics and associated probabilities of obtaining a result more extreme than the ones observed (*p* values) are provided for each setup.

Sample (PDB code)	1ier		4f5s		1wla	
Concentration (mg ml^−1^)	0.5	2.0	0.5	2.0	0.5	2.0
Detector distance (m)	6.0		3.0		1.5	
Angular range (nm^−1^)	[0.0145–3.8497]		[0.0291–7.6499]		[0.0581–14.9253]	
χ^2^/(*n* − 1)	1.026	0.993	0.997	0.975	1.048	0.958
*p*(>χ^2^)	0.172	0.600	0.533	0.816	0.042	0.940
Anderson–Darling *A* ^2^	2.771	0.530	1.932	1.414	0.742	2.548
*p*(>*A* ^2^)	0.0359	0.716	0.100	0.198	0.525	0.047

## Footnotes

1 Namely 0.01632exp[10exp(100*s*)], where 0.01632 corresponds to the expected scattering of water at 293 K. The scaling factors 10 and 100 were empirically determined.
